# Association between DASH diet adherence and mortality in non-diabetic adults with and without chronic kidney disease

**DOI:** 10.1186/s12986-026-01118-z

**Published:** 2026-04-09

**Authors:** Shang-Feng Tsai, Wei‑Ju Liu, Yu-Jung Lin, Chia-Lin Lee

**Affiliations:** 1 Department of Post-Baccalaureate Medicine, College of Medicine, National Chung Hsing University, 402202 Taichung, Taiwan; 2 Division of Nephrology, Department of Internal Medicine, Taichung Veterans General Hospital, 40705 Taichung, Taiwan; 3 Clinical Informatics Division, Department of Digital Medicine, Taichung Veterans General Hospital, 40705 Taichung, Taiwan; 4 Intelligent Data Mining Laboratory, Department of Medical Research, Taichung Veterans General Hospital, 40705 Taichung, Taiwan; 5 Division of Endocrinology and Metabolism, Department of Internal Medicine, Taichung Veterans General Hospital, 40705 Taichung, Taiwan; 6 Division of Artificial Intelligence, Department of Digital Medicine, Taichung Veterans General Hospital, Taichung, 40705 Taiwan; 7 School of Medicine, College of Medicine, National Yang Ming Chiao Tung University, Taipei, 112304 Taiwan

**Keywords:** Dietary approaches to stop hypertension (DASH) diet, Chronic kidney disease (CKD), Mortality, National health and nutrition examination survey (NHANES)

## Abstract

**Background:**

The Dietary Approaches to Stop Hypertension (DASH) diet has been associated with reduced cardiovascular risk, but its impact on mortality in individuals with chronic kidney disease (CKD) remains uncertain.

**Methods:**

We analyzed data from 37,827 adults in the NHANES 1999–2014 cycles (without diabetes). Participants were stratified by CKD status (eGFR < 60 mL/min/1.73 m²) and DASH adherence (above or below median score). Mortality outcomes were assessed via the National Death Index. Multivariable Cox regression models, adjusted for demographics, comorbidities, and diet, were used to evaluate associations with all-cause, cardiovascular (CV), and cancer mortality.

**Results:**

High DASH adherence was associated with healthier metabolic profiles and lower blood pressure. Among non-CKD participants, higher DASH scores were linked to significantly lower composite CV or cancer mortality (HR 0.896; 95% CI, 0.806–0.997; *p* = 0.0431), but not to all-cause, CV, or cancer mortality individually. No significant mortality associations were observed in participants with CKD, and interaction effects between DASH and CKD status were non-significant. Higher intake of dietary fiber, magnesium, and potassium was associated with reduced mortality in the overall and non-CKD populations. However, in CKD participants, high magnesium and potassium intake were paradoxically associated with increased cancer mortality.

**Conclusion:**

Greater adherence to the DASH diet is associated with lower CV and cancer-related mortality among individuals without CKD. In CKD patients, the benefits are less clear, and some nutrients may have differential effects. Personalized dietary strategies based on kidney function may be warranted.

**Supplementary Information:**

The online version contains supplementary material available at 10.1186/s12986-026-01118-z.

## Lay summary

This study explored whether the DASH diet, known for lowering blood pressure, could reduce the risk of death in people with and without chronic kidney disease (CKD). Researchers analyzed data from 37,827 U.S. adults collected between 1999 and 2014. Participants were grouped by kidney function and how closely they followed the DASH diet. Among those without CKD, higher DASH adherence was linked to lower risk of death from heart disease or cancer. However, in people with CKD, no clear benefit was observed, and higher intakes of magnesium and potassium—normally healthy—were linked to increased cancer risk. This suggests that while the DASH diet may help many people live longer, those with CKD may need a more personalized diet. The findings highlight the importance of adjusting dietary recommendations based on kidney function.

**Supplementary Information:**

The online version contains supplementary material available at 10.1186/s12986-026-01118-z.

## Introduction

Chronic kidney disease (CKD) is a major global health burden, affecting approximately 10–15% of the worldwide population [1]. Patients with CKD face an increased risk of progression to end-stage kidney disease requiring dialysis, as well as higher mortality, particularly from cardiovascular (CV) causes [2]. According to the 2024 Kidney Disease: Improving Global Outcomes (KDIGO) guidelines [3], in addition to pharmacologic therapies such as renin–angiotensin system inhibitors [4–6], sodium–glucose co-transporter 2 inhibitors [7, 8], and finerenone [9, 10] and GLP-1 receptor agonists [11], lifestyle modification (including dietary approaches) (from 2020 KDOQI guideline) [12] remains a cornerstone of CKD management. These modifications include dietary intervention and physical activity. Since 2016, a landmark study published in the Journal of the American Society of Nephrology by Garneata et al. [13]. demonstrated that a very low-protein diet (VLPD) supplemented with ketoanalogues was effective in slowing renal function decline in non-diabetic CKD patients. More recently, another study demonstrated that a LPD reduces proteinuria and slows the decline in glomerular filtration rate (GFR) in patients with DKD [14]. Therefore, a LPD or VLPD has been considered a cornerstone of dietary intervention in patients with CKD. However, other dietary patterns, such as the Dietary Approaches to Stop Hypertension (DASH) and the Mediterranean diet, have also been investigated.

The DASH diet, initially designed for the general population to improve blood pressure (BP) control and metabolic health, has been associated with reduced CV mortality in general population [15–17]. The dash diet pattern focused on “vegetables and fruits” diet. However, its use in CKD patients remains controversial [18]. Concerns include limited renal [18–20] and further limited CV outcome data [18, 21], potential conflicts with current CKD dietary guidelines [22]—particularly regarding protein, potassium, and phosphate intake—and the potential risk of hyperkalemia. Current evidence on the impact of the DASH diet on mortality includes one study in hemodialysis patients [20] and one in early-stage CKD [21], but none specifically in patients with an eGFR below 60 mL/min/1.73 m². The 2012 KDOQI update [23] attempted to reconcile some of these issues, with some experts advocating stricter sodium restriction (e.g., < 1.5 g/day) and balanced macronutrient distribution. Current clinical practice guidelines for CKD do not support the routine implementation of the DASH diet in patients with moderate renal impairment [3, 24].

In this study, we utilized data (non-diabetic patients) from the National Health and Nutrition Examination Survey (NHANES), which includes a validated dietary questionnaire, to evaluate the associations between DASH diet adherence and various mortality outcomes—including all-cause, CV, and cancer mortality—in participants with and without CKD (GFR < 60 mL/min/1.73 m²). Additionally, we explored the impact of individual DASH components on these outcomes to determine whether specific dietary elements may differentially influence mortality risk. To avoid confounding from diabetic kidney disease, individuals with diagnosed diabetes were excluded from the present analysis. Therefore, all CKD participants represent non-diabetic CKD.

## Materials and methods

### Study population and data collection

#### National health and nutrition examination survey (NHANES)

The National Health and Nutrition Examination Survey (NHANES) is a series of health-related programs conducted periodically by the National Center for Health Statistics (NCHS), a division of the Centers for Disease Control and Prevention (CDC) in the United States. NHANES data are publicly available and widely used in epidemiological research. The survey protocol was approved by the NCHS Research Ethics Review Board, and all participants or their proxies provided written informed consent prior to participation.

**Fig. 1:**
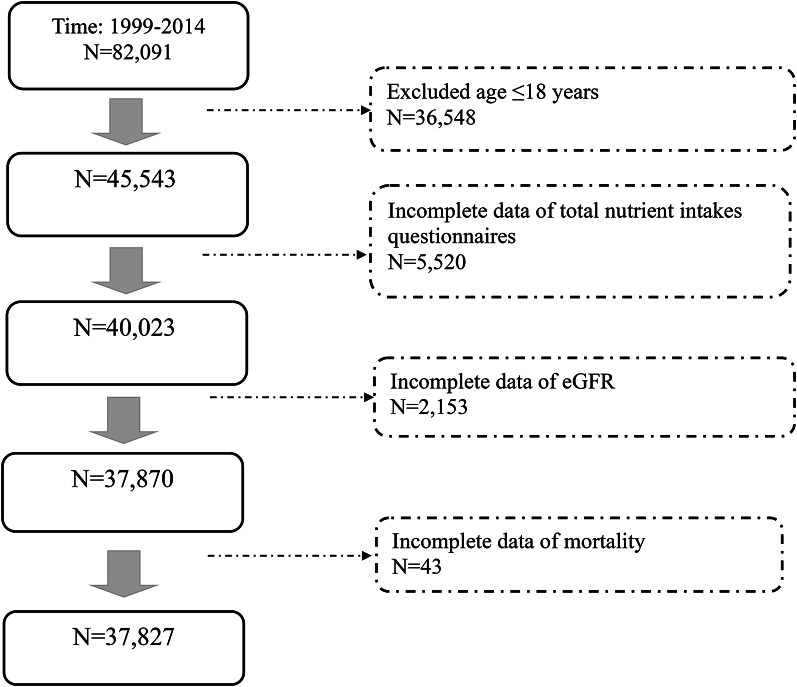
Patient selection algorithm

NHANES is a large, cross-sectional survey designed to evaluate the health and nutritional status of the non-institutionalized civilian population in the United States. It combines data from in-home interviews, physical examinations, anthropometric assessments, health and dietary questionnaires, and laboratory tests. Figure 1 presents the flowchart of participant selection for the present study. We included adult participants from NHANES cycles between 1999 and 2014. Individuals were excluded if they were aged ≤ 18 years, had incomplete data on total nutrient intake, missing Chronic Kidney Disease Epidemiology Collaboration (CKD-EPI) for Glomerular Filtration Rate (GFR), or missing mortality data.

Information on diabetes mellitus (DM) and coronary heart disease (CHD) was obtained via structured questionnaires. Specifically, CHD was identified using MCQ160C, cancer using MCQ220, and DM using DIQ010, as defined by NHANES documentation (https://wwwn.cdc.gov/Nchs/Nhanes/2009-2010/MCQ_F.htm#MCQ160C). Mortality status was ascertained through probabilistic record linkage with the National Death Index (NDI), a comprehensive registry of death certificate data for U.S. residents since 1979. The NHANES Public-use Linked Mortality Files (https://wwwn.cdc.gov/nchs/nhanes/ContinuousNhanes/Default.aspx?BeginYear=2009) provided follow-up data on vital status and causes of death. Causes of death were classified using the UCOD_LEADING variable into all-cause mortality, cardiovascular mortality (UCOD_LEADING codes 001: Diseases of heart and 005: Cerebrovascular diseases), and cancer mortality (UCOD_LEADING code 002: Malignant neoplasms).

### Definition of nutrition

Dietary intake was assessed using 24-hour dietary recalls conducted by trained interviewers using the USDA Automated Multiple-Pass Method. During each NHANES cycle, participants completed at least one in-person 24-hour recall at the Mobile Examination Center; in later cycles, a second recall was obtained via telephone. Nutrient intakes were calculated based on these recalls and used to derive DASH adherence scores according to established scoring algorithms. For consistency across cycles, we used the first-day dietary recall, which is available for all participants.

The DASH score was calculated according to the nutrient-based scoring system described by Mellen et al. [25]. This approach evaluates nine target nutrients reflective of the DASH dietary pattern: total fat, saturated fat, protein, cholesterol, fiber, magnesium, calcium, potassium, and sodium. For each nutrient, participants received a score of 1 if the intake met the DASH target, 0.5 if it met an intermediate target, and 0 otherwise. The total DASH score ranged from 0 to 9, with higher scores indicating greater adherence to the DASH dietary pattern (Supplementary Table 1).

### Other data collection

Baseline variables included age, sex, body mass index (BMI) (kg/m^2^), eGFR calculated using the CKD-EPI equation (mL/min/1.73 m²) [26], race/ethnicity (categorized as non-Hispanic White, non-Hispanic Black, and Mexican American/Other), systolic and diastolic blood pressure (SBP and DBP, mmHg), total cholesterol (TC), high-density lipoprotein cholesterol (HDL-C) (mg/dL), fasting plasma glucose (mg/dL), and glycated hemoglobin (HbA1c, %). Daily caloric intake (kcal/day) was recorded along with the percentage contribution of carbohydrates, fats, and proteins. Dietary quality was assessed using the Dietary Approaches to Stop Hypertension (DASH) and alternate Mediterranean diet (aMED) scores. Chronic kidney disease (CKD) was defined as an eGFR < 60 mL/min/1.73 m².

The present study is a secondary analysis of publicly available, de-identified data from the NHANES. NHANES protocols were approved by the National Center for Health Statistics (NCHS) Research Ethics Review Board, and all participants provided written informed consent. The current analysis was reviewed and approved by the Institutional Review Board of Taichung Veterans General Hospital, Taiwan (IRB No. CE18312A).

### Statistical analyses

Participants were stratified based on the presence or absence of CKD, and by adherence to the DASH diet, categorized as below or above the median DASH score by Weighted Cox proportional hazards regression models. Multivariable analyses were conducted to examine the association between DASH adherence and various mortality outcomes, including all-cause mortality, cardiovascular (CV) mortality, cancer mortality, and combined CV or cancer mortality. These analyses were adjusted for age, sex, BMI, race/ethnicity, hypertension status, and daily energy intake. In addition, subgroup analyses were performed separately for participants with and without CKD. Finally, we assessed the joint effects of baseline CKD status and DASH adherence on mortality outcomes. The proportional hazards assumption for the Cox regression models was assessed using Schoenfeld residuals and by testing time-dependent covariate interactions with log(time) [27]. No significant violations of the proportional hazards assumption were observed for the main exposure or other covariates.

All statistical analyses were performed using SAS version 9.2 (SAS Institute Inc., Cary, NC, USA) and SUDAAN version 11.0 (Research Triangle Institute, Research Triangle Park, NC, USA). Survey sample weights were applied to account for the complex, multistage probability sampling design of NHANES. Data are presented as means ± standard errors (SE), unless otherwise specified.

## Results

### Participant inclusion and exclusion criteria

The patient selection algorithm is illustrated in Fig. 1. A total of 82,091 participants were enrolled in NHANES between 1999 and 2014. After excluding individuals aged ≤ 18 years (*n* = 36,548), 45,543 adults remained eligible. Of these, 5,520 participants were excluded due to incomplete dietary intake questionnaire data, and an additional 2,153 were excluded due to missing eGFR data. Lastly, 43 participants with incomplete mortality data were excluded. The final analytic sample comprised 37,827 participants. Using the scoring method developed by Mellen et al. [25], the median DASH diet adherence score was 2.

### Characteristics of study participants according to their baseline renal function (CKD or not)

Table 1 presents the baseline characteristics of the 37,827 participants, stratified by CKD status. Among them, 3,140 participants (8.3%) had CKD, defined as an eGFR < 60 mL/min/1.73 m², while 34,687 did not have CKD. Participants with CKD were significantly older than those without CKD (70.9 vs. 44.6 years, *p* < 0.0001) and had a lower proportion of males (41.2% vs. 49.0%, *p* < 0.0001). The CKD group had a higher prevalence of non-Hispanic White individuals (82.1% vs. 69.6%) and a lower proportion of Mexican American or other ethnicities (8.6% vs. 19.8%, *p* < 0.0001). CKD participants exhibited significantly higher systolic blood pressure (136.9 vs. 121.8 mmHg) and lower diastolic blood pressure (66.1 vs. 71.4 mmHg). The prevalence of hypertension was substantially higher in the CKD group (69.6% vs. 26.7%, *p* < 0.0001). Metabolic parameters also differed between groups. Participants with CKD had higher BMI (29.3 vs. 28.5 kg/m²), fasting plasma glucose (111.0 vs. 96.5 mg/dL), and HbA1c levels (6.0% vs. 5.5%, all *p* < 0.0001). They also had higher triglyceride levels and lower total calorie intake (1708.7 vs. 2242.6 kcal/day). Regarding dietary patterns, the CKD group had slightly lower DASH scores (2.28 vs. 2.39, *p* < 0.0001) but slightly higher aMED scores (3.54 vs. 3.40, *p* < 0.0001), suggesting subtle differences in dietary adherence between groups.

**Table 1:** Characteristics of study participants according to their baseline renal function (CKD or not)

Variables	Overall	CKD (-)	CKD (+)	*p*
Number of participants	37,827	34,687	3140	
Age (years old)	46.24(45.82–46.66)	44.57(44.18–44.96)	70.94(70.33–71.56)	< 0.0001
Male, n (%)	18,344(48.47)	16,846(48.96)	1498(41.19)	< 0.0001
Race/ethnicity				
Non-Hispanic white	17,849(70.39)	15,810(69.6)	2039(82.07)	< 0.0001
Non-Hispanic black	7570(10.51)	6986(10.58)	584(9.36)	
Mexican American/other	12,408(19.11)	11,891(19.82)	517(8.57)	
Body mass index (kg/m^2^)	28.5(28.37–28.63)	28.45(28.31–28.58)	29.31(29.04–29.58)	< 0.0001
Systolic blood pressure (mm Hg)	122.71(122.32–123.1)	121.77(121.39-122.16)	136.9(135.77-138.04)	< 0.0001
Diastolic blood pressure (mm Hg)	71.03(70.72–71.34)	71.36(71.06–71.66)	66.05(65.19–66.91)	< 0.0001
Hypertension, n (%)	12,333(29.38)	10,074(26.66)	2259(69.59)	< 0.0001
Total cholesterol (mg/dl)	197.69(196.97–198.4)	197.84(197.11-198.58)	195.34(192.95-197.74)	< 0.0001
HDL cholesterol (mg/dl)	52.8(52.48–53.12)	52.81(52.49–53.14)	52.52(51.71–53.34)	< 0.0001
Triglycerides (mg/dl)	150.25(148.04-152.45)	148.98(146.66-151.29)	169.07(164.15-173.98)	< 0.0001
Fasting plasma glucose (mg/dl)	97.37(96.9-97.83)	96.45(95.98–96.92)	110.95(108.93-112.96)	< 0.0001
HbA1c (%)	5.53(5.52–5.55)	5.5(5.49–5.52)	5.98(5.93–6.02)	< 0.0001
eGFR (mL/min/1.73 m^2^)	96.17(95.59–96.75)	99.48(98.97–99.98)	47.16(46.62–47.7)	< 0.0001
Daily calories (kcal/day)	2208.83(2193.75-2223.91)	2242.59(2227.39-2257.79)	1708.74(1674.77-1742.71)	< 0.0001
% from carbohydrate	49.94(49.73–50.15)	49.94(49.73–50.16)	49.85(49.33–50.37)	< 0.0001
% from fat	34.16(33.98–34.34)	34.17(33.98–34.35)	34.13(33.76–34.51)	< 0.0001
% from protein	15.9(15.8–16)	15.89(15.79–15.99)	16.02(15.74–16.3)	< 0.0001
DASH score	2.38(2.36–2.41)	2.39(2.36–2.42)	2.28(2.23–2.34)	< 0.0001
aMED	3.4(3.36–3.44)	3.4(3.35–3.44)	3.54(3.46–3.62)	< 0.0001

Data are presented as mean (95% CI) or n (%). aMED, alternative Mediterranean Diet Index. DASH, Dietary Approaches to Stop Hypertension. eGFR, estimated glomerular filtration rate. HbA1c, glycated hemoglobin. HDL, high-density lipoprotein

### Characteristics of study participants according to their DASH Score

Baseline characteristics of participants stratified by adherence to the DASH diet are shown in Table 2. Among the 37,827 participants, 19,526 (51.6%) had DASH scores below the median (*≤* 2), while 18,301 (48.4%) had scores above the median. Participants with higher DASH scores were less likely to be male (45.9% vs. 51.3%, *p* < 0.0001) and more likely to be of non-Hispanic White ethnicity (70.9% vs. 69.9%). They had lower BMI (28.1 vs. 28.9 kg/m²), lower systolic and diastolic blood pressure, and a lower prevalence of hypertension (*p* < 0.0001 for all comparisons). Metabolically, those with higher DASH adherence had significantly lower triglyceride levels (148.9 vs. 151.8 mg/dL), lower fasting plasma glucose (96.98 vs. 97.72 mg/dL), and lower HbA1c levels (5.52% vs. 5.55%, all *p* < 0.0001). eGFR levels were slightly higher in the high DASH group (96.25 vs. 96.09 mL/min/1.73 m², *p* < 0.0001), suggesting better renal function. Nutritionally, participants with higher DASH scores had higher daily caloric intake (2,292 vs. 2,132 kcal/day), with a higher percentage of calories from carbohydrates and a lower percentage from fat and protein. Moreover, they had significantly higher aMED scores (3.61 vs. 1.26, *p* < 0.0001), indicating generally healthier dietary patterns. We found no significant difference in baseline serum albumin levels between the DASH score groups [4.0 (3.67–4.25) vs. 3.9 (3.70–4.35); *p* = 0.8631], indicating comparable visceral protein status at baseline.

**Table 2:** Characteristics of study participants according to the DASH score

	DASH score (median = 2)		*P﻿*
	*≤*median	> median	
Number of participants	19,526	18,301	
Age (years old)	46.22(45.75–46.69)	46.26(45.79–46.73)	< 0.0001
Male, n (%)	9062(45.9)	9282(51.28)	
Race/ethnicity			
Non-Hispanic white	9398(70.87)	8451(69.86)	< 0.0001
Non-Hispanic black	4490(12.11)	3080(8.76)	
Mexican American/other	5638(17.02)	6770(21.38)	
Body mass index (kg/m^2^)	28.9(28.74–29.06)	28.07(27.92–28.22)	< 0.0001
Serum albumin (g/dl)	4.0 (3.67–4.25)	3.9 (3.70–4.35)	0.8631
Systolic blood pressure (mm Hg)	122.87(122.42-123.32)	122.54(122.1-122.98)	< 0.0001
Diastolic blood pressure (mm Hg)	71.23(70.87–71.58)	70.82(70.48–71.17)	< 0.0001
Hypertension, n (%)	6511(30.19)	5822(28.49)	0.0064
Total cholesterol (mg/dl)	197.97(197.17-198.77)	197.38(196.42-198.34)	< 0.0001
HDL cholesterol (mg/dl)	52.64(52.24–53.04)	52.97(52.57–53.37)	< 0.0001
Triglycerides (mg/dl)	148.86(145.7-152.03)	151.76(149.02-154.49)	< 0.0001
Fasting plasma glucose (mg/dl)	97.72(97.11–98.33)	96.98(96.36–97.61)	< 0.0001
HbA1c (%)	5.55(5.53–5.56)	5.52(5.5–5.54)	< 0.0001
eGFR (mL/min/1.73 m^2^)	96.09(95.41–96.78)	96.25(95.65–96.85)	< 0.0001
Daily calories (kcal/day)	2132.35(2115.85-2148.85)	2292.23(2266.81-2317.65)	< 0.0001
% from carbohydrate	46.79(46.57–47.01)	53.37(53.08–53.65)	< 0.0001
% from fat	37.89(37.72–38.05)	30.1(29.86–30.34)	< 0.0001
% from protein	15.32(15.22–15.43)	16.53(16.4-16.66)	< 0.0001
DASH score	1.26(1.24–1.27)	3.61(3.59–3.64)	< 0.0001
aMED	19,526	18,301	< 0.0001

Data are presented as mean (95% CI) or n (%). DASH, Dietary Approaches to Stop Hypertension. eGFR, estimated glomerular filtration rate. HbA1c, glycated hemoglobin. HDL, high-density lipoprotein

### Association between DASH score and mortality

The median follow-up time was 11.5 years (interquartile range, 7.9–16.0), corresponding to a total of 436385.3 person-years of follow. The associations between adherence to the DASH diet and various mortality outcomes are presented in Table 3. After adjusting for age, sex, BMI, race/ethnicity, hypertension status, and daily energy intake, higher DASH adherence was associated with a trend toward reduced all-cause mortality in participants without CKD (adjusted HR 0.935; 95% CI, 0.868–1.008; *p* = 0.0783), although this did not reach statistical significance. A similar non-significant association was observed in participants with CKD (HR 0.956; 95% CI, 0.852–1.072; *p* = 0.4391). The interaction between DASH adherence and CKD status for all-cause mortality was not significant (*p* for interaction = 0.7652). For CV mortality, no significant associations were observed in either subgroup (CKD–: HR 0.908, 95% CI: 0.785–1.051, *p* = 0.1942; CKD+: HR 0.940, 95% CI: 0.783–1.128, *p* = 0.5038). Similarly, no significant associations were found between DASH adherence and cancer mortality in both CKD– (HR 0.883, 95% CI: 0.752–1.037, *p* = 0.1279) and CKD+ groups (HR 0.998, 95% CI: 0.751–1.327, *p* = 0.9895). The interaction *p*-value for cancer mortality was 0.4149. Notably, higher DASH adherence was significantly associated with a reduced risk of composite CV or cancer mortality in participants without CKD (HR 0.896; 95% CI, 0.806–0.997; *p* = 0.0431), but not in those with CKD (HR 0.957; 95% CI, 0.816–1.121; *p* = 0.581). However, the interaction effect between DASH adherence and CKD status was not statistically significant (*p* for interaction = 0.4980).

**Table 3:** Associations of adherence to DASH with mortality

	High DASH adherence (≥ median) (Event number/all number)	Low DASH adherence (< median) (Event number/all number)	Adjusted HR (95% CI)^a^	P	P for interaction
	**All-cause mortality**				
Overall population					
CKD (-)	2255/34687	2484/34687	0.935(0.868-1.008)	0.0783	0.7652
CKD (+)	863/3140	1012/3140	0.956(0.852-1.072)	0.4391	
	**CV mortality**				
Overall population					
CKD (-)	651/34687	743/34687	0.908(0.785-1.051)	0.1942	0.7841
CKD (+)	327/3140	376/3140	0.94(0.783-1.128)	0.5038	
	**Cancer mortality**				
Overall population					
CKD (-)	537/34687	618/34687	0.883(0.752-1.037)	0.1279	0.4149
CKD (+)	139/3140	158/3140	0.998(0.751-1.327)	0.9895	
	**CV or Cancer mortality**				
Overall population					
CKD (-)	1188/34687	1361/34687	0.896(0.806-0.997)	0.0431	0.4980
CKD (+)	466/3140	534/3140	0.957(0.816-1.121)	0.581	

DASH, Dietary Approaches to Stop Hypertension. a Adjusted for age, gender, body mass index, race, hypertension, and daily energy intake

Participants were bifurcated into groups based on the median DASH score of 2

### Joint effects of DASH score and CKD status on mortality

**Fig. 2:**
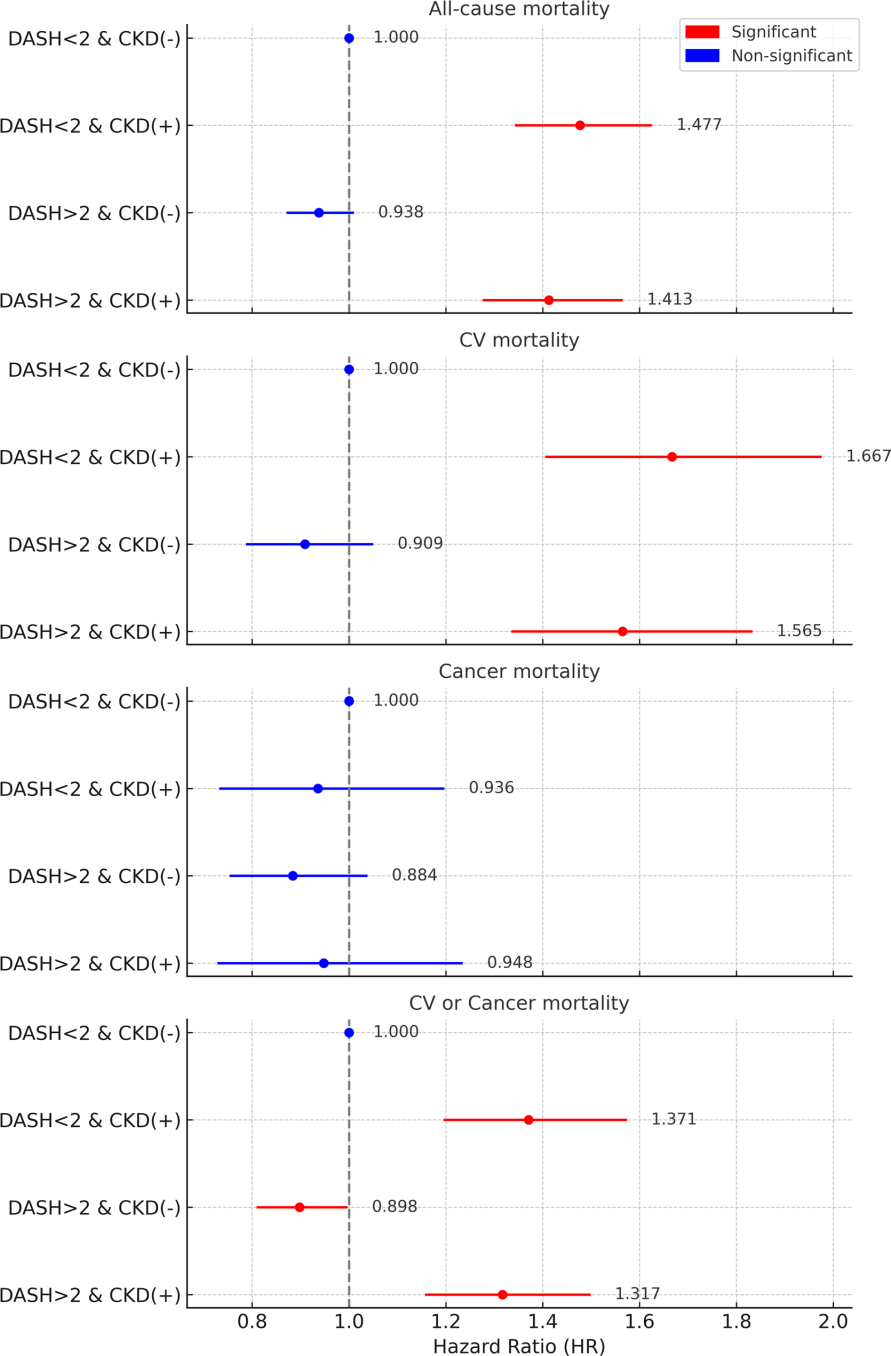
Joint effects of DASH and baseline CKD status on all mortality

The joint associations of adherence to the DASH diet and baseline CKD status with various mortality outcomes are summarized in Supplementary Tables 2 and illustrated in Fig. 2. For all-cause mortality, participants with CKD and low DASH scores (< 2) had the highest risk (HR 1.477; 95% CI, 1.342–1.626; *p* < 0.0001) compared to the reference group (non-CKD with low DASH score). Those with CKD and high DASH scores also showed significantly increased mortality risk (HR 1.413; 95% CI, 1.276–1.565; *p* < 0.0001), while non-CKD participants with high DASH scores had a non-significant trend toward reduced risk (HR 0.938; 95% CI, 0.871–1.010; *p* = 0.0877). The interaction between DASH and CKD status was not statistically significant (*p* for interaction = 0.7652). For CV, a similar pattern was observed. CKD participants with low DASH scores had a markedly elevated risk (HR 1.667; 95% CI, 1.405–1.976; *p* < 0.0001), followed by those with CKD and high DASH scores (HR 1.565; 95% CI, 1.335–1.833; *p* < 0.0001). No significant reduction in risk was observed for non-CKD participants with high DASH scores (HR 0.909; 95% CI, 0.787–1.050; *p* = 0.1934; *p* for interaction = 0.7841). For cancer mortality, no statistically significant associations were found across any of the subgroups. The HRs for CKD and non-CKD participants with high DASH scores were 0.948 (95% CI, 0.728–1.235) and 0.884 (95% CI, 0.753–1.038), respectively. For composite CV or cancer mortality, participants with CKD and low DASH scores again had the highest risk (HR 1.371; 95% CI, 1.195–1.574; *p* < 0.0001). Participants without CKD but with high DASH adherence exhibited a significantly lower risk (HR 0.898; 95% CI, 0.809–0.997; *p* = 0.0443). The interaction p-value was not significant (*p* = 0.4980).

Overall, the joint effect analysis demonstrated that CKD was associated with elevated mortality risk regardless of DASH score, and that high DASH adherence may attenuate risk in individuals without CKD, particularly for CV or cancer-related death.

### Association between individual DASH diet components and cardiovascular mortality

Supplementary Table 3 presents the associations between individual components of the DASH dietary pattern and CV mortality among NHANES participants, stratified by CKD status. No significant associations were observed for saturated fat, total fat, protein, or cholesterol intake with CV mortality in any group. Among all participants, higher dietary fiber intake was significantly associated with lower CV mortality. Compared to the lowest intake group (score = 0), those with moderate (0.5) and high (1) fiber intake had reduced risks (HR 0.739, 95% CI: 0.568–0.961, *p* = 0.0245; and HR 0.697, 95% CI: 0.513–0.946, *p* = 0.0212, respectively). Similar trends were observed in participants without CKD, with significant association at the highest fiber level (HR 0.699, 95% CI: 0.497–0.984, *p* = 0.0401). Magnesium intake was also significantly associated with lower CV mortality in both the overall population and subgroups. In the overall population, moderate magnesium intake was associated with the greatest reduction in risk (HR 0.571, 95% CI: 0.428–0.762, *p* = 0.0002). This association remained significant in both CKD– (HR 0.610, *p* = 0.0024) and CKD+ groups (HR 0.442, *p* = 0.0491). For potassium, high intake (score = 1) was significantly associated with lower CV mortality in the overall population (HR 0.718, 95% CI: 0.563–0.914, *p* = 0.0077) and in those without CKD (HR 0.743, 95% CI: 0.568–0.972, *p* = 0.0306). Conversely, high sodium intake was associated with increased CV mortality in the non-CKD group (HR 1.324, 95% CI: 1.034–1.695, *p* = 0.0266), though not in the overall population or among those with CKD. The interaction p-value for sodium approached statistical significance (*p* = 0.0507), suggesting a possible differential effect by CKD status.

### Association between individual DASH diet components and cancer mortality

Supplementary Table 4 displays the associations between individual dietary components of the DASH diet and cancer mortality among NHANES participants, stratified by CKD status.

Among all participants, higher intake of dietary fiber was significantly associated with a reduced risk of cancer mortality. Compared with the lowest intake group (score = 0), those with moderate (0.5) and high (1) fiber intake had lower cancer mortality risks (HR 0.676; 95% CI: 0.513–0.889; *p* = 0.0056 and HR 0.564; 95% CI: 0.407–0.781; *p* = 0.0007, respectively). This association remained significant in participants without CKD. In contrast, no benefit was observed in those with CKD. Magnesium intake also showed an inverse association with cancer mortality. In the overall cohort, moderate intake (score = 0.5) was associated with reduced risk (HR 0.591; 95% CI: 0.448–0.779; *p* = 0.0003). This effect persisted among non-CKD participants (HR 0.558; 95% CI: 0.416–0.748; *p* = 0.0001), but not in those with CKD. Notably, among CKD patients with high magnesium intake (score = 1), cancer mortality risk was significantly elevated (HR 2.285; 95% CI: 1.256–4.156; *p* = 0.0073; p for interaction = 0.0023). Similarly, potassium intake showed a protective association in the general and non-CKD populations. Moderate intake was linked to reduced cancer mortality (HR 0.738; *p* = 0.0273 overall; HR 0.686; *p* = 0.0067 in non-CKD), while high potassium intake among CKD participants was associated with an increased risk (HR 1.841; 95% CI: 1.029–3.296; *p* = 0.0401; p for interaction = 0.0123). In contrast, high sodium intake (score = 1) was associated with increased cancer mortality in the overall population (HR 1.367; 95% CI: 1.131–1.654; *p* = 0.0015) and in non-CKD individuals (HR 1.399; *p* = 0.0028). No significant association was observed in the CKD group. Other components such as saturated fat, total fat, protein, cholesterol, and calcium showed no consistent or significant associations with cancer mortality across strata.

### Association between individual DASH diet components and all-cause mortality

Supplementary Table 5 summarizes the associations between individual components of the DASH diet and all-cause mortality among NHANES participants. In the overall population, higher intake of dietary fiber was consistently associated with lower all-cause mortality. Compared to the reference group (score = 0), participants with moderate and high fiber intake had significantly reduced risks (HR 0.714, 95% CI: 0.627–0.814, *p* < 0.0001; and HR 0.679, 95% CI: 0.602–0.765, *p* < 0.0001, respectively). These associations remained significant in participants without CKD but were not observed in those with CKD (p for interaction = 0.0221). Magnesium intake was similarly associated with lower all-cause mortality. Moderate intake (score = 0.5) yielded an HR of 0.683 (95% CI: 0.588–0.793, *p* < 0.0001), and high intake (score = 1) was also protective (HR 0.766; *p* = 0.0002). These findings were consistent in the non-CKD population but not significant among CKD participants. Potassium intake was inversely associated with all-cause mortality in the overall and non-CKD populations (HR 0.805 and 0.794, respectively; both *p* < 0.005), but not in those with CKD. In contrast, sodium intake showed a positive association with mortality. Participants with high sodium intake (score = 1) had significantly increased risk in the overall population (HR 1.322; 95% CI: 1.199–1.458; *p* < 0.0001) and in the non-CKD group (HR 1.371; *p* < 0.0001), but this was not observed in the CKD subgroup. Saturated fat intake at moderate levels was inversely associated with mortality in the overall and non-CKD populations (HR ~ 0.89, *p* < 0.03), though not at high intake levels. Interestingly, higher protein intake (score = 1) was associated with lower mortality in CKD participants (HR 0.828; *p* = 0.0233), but not in the non-CKD group (p for interaction = 0.1987). No significant associations were observed for total fat, cholesterol, or calcium intake across groups.

## Discussion

This is the first study to evaluate the effects of overall DASH adherence and its individual dietary components on mortality outcomes, stratified by CKD status. In participants without CKD, higher adherence to the DASH diet was associated with a reduced risk of composite CV or cancer mortality. However, no significant associations were observed in those with CKD. Analysis of individual DASH components revealed that higher intake of dietary fiber, magnesium, and potassium was consistently linked to lower CV, cancer and call-cause mortality risk, particularly among non-CKD participants. In contrast, high sodium intake was associated with increased mortality. Notably, among individuals with CKD, higher intakes of magnesium and potassium were paradoxically associated with increased cancer mortality. This study suggests that the mortality benefit associated with DASH adherence in the general population was not clearly observed in individuals with pre-existing moderate CKD. The observed outcomes were not associated with baseline nutritional status, given the comparable baseline serum albumin levels across groups and the statistical adjustment for baseline BMI.

The DASH diet was originally developed to reduce BP [28], and has also been associated with reduced CV risk and all-cause mortality in the general population [15–17]. However, studies evaluating its effects in patients with renal dysfunction remain limited [20, 21, 29]. According to a previous NHANES-based study [20], poor adherence to the DASH dietary pattern was associated with a higher risk of ESKD among adults with moderate chronic kidney disease and hypertension. Compared to our analysis, that study utilized an earlier NHANES cohort (1988–1994 vs. 1999–2014) and focused on renal outcomes rather than survival. A meta-analysis suggested that the DASH diet may reduce mortality in patients with early CKD [21] ; however, all included participants had preserved kidney function (eGFR > 60 mL/min/1.73 m²). In the DIET-HD multinational cohort study [29], neither the Mediterranean nor DASH dietary patterns were associated with CV or all-cause mortality among patients receiving hemodialysis. In contrast to these previous two studies [21, 29], our study is the first to specifically examine the association between DASH adherence and mortality in patients with moderate CKD.

There are possible explanations for why adherence to the DASH diet has been associated with reduced CV and all-cause mortality in individuals with early-stage CKD [21], but not in patients with advanced CKD in our study or those undergoing hemodialysis, as shown in the DIET-HD study [29]. First, in early CKD, CV mortality is primarily driven by atherosclerotic processes over intima resulting from metabolic disorders. However, as kidney function declines, a shift in pathophysiology occurs—novel and uremia-related risk factors contribute to vascular damage [30], predominantly through medial arterial calcification rather than intimal atherosclerosis [31, 32]. This phenomenon also explains why statins, which target lipid-driven atherosclerosis, failed to improve CV outcomes in ESKD populations [33], as demonstrated in the SHARP [34], 4D [35], and AURORA [36] trials. Similarly, the DASH diet may not effectively counteract the uremia-driven vascular pathology observed in advanced kidney disease. Second, several key components of the DASH diet—particularly sodium, potassium, and magnesium—may act as confounding factors. In patients with advanced CKD, high intake of these electrolytes is often contraindicated due to concerns about volume overload [37], hyperkalemia [38], and hypermagnesemia [39, 40], which may increase mortality risk. Our findings support this notion, suggesting that any potential mortality benefit of the DASH diet may be offset or negated by the risks associated with higher intake of sodium, potassium, and magnesium in this population. This may partly explain the limited applicability and existing concerns regarding the DASH diet in patients with advanced CKD [18]. Third, the absence of a statistically significant interaction may reflect limited statistical power within the CKD subgroup, given its smaller sample size and higher baseline mortality rate. Therefore, rather than concluding that DASH is ineffective in CKD, our findings indicate that a measurable protective signal was not detectable in this population under current analytic conditions. Fourth, patients with CKD carry a substantially elevated baseline risk of mortality driven by renal dysfunction itself. Even if DASH adherence confers modest cardiometabolic benefits, these effects may be relatively small compared to the magnitude of CKD-associated risk. In such a high-risk population, the incremental benefit of dietary modification may be diluted or overwhelmed by residual uremic risk, including neurohormonal activation, chronic inflammation, anemia, mineral bone disorder, and vascular calcification. Consequently, the net observable association between DASH adherence and mortality may appear neutral. Fifth, competing risks may further attenuate observable dietary effects in CKD. Mortality in CKD is influenced by multiple non-atherosclerotic mechanisms, including infection, sudden cardiac death, arrhythmia, and progressive heart failure. These competing pathways may limit the relative contribution of diet-related atherosclerotic modification. Moreover, CKD populations often exhibit “reverse epidemiology,” wherein traditional cardiometabolic risk factors do not behave in a conventional manner. Nutritional restriction in advanced CKD may inadvertently worsen protein-energy wasting, potentially offsetting benefits derived from improved dietary quality. Finally, a further explanation lies in the well-described malnutrition–inflammation–atherosclerosis (MIA) syndrome in CKD [41]. In advanced renal dysfunction, chronic inflammation [42], oxidative stress, and protein-energy wasting form a self-perpetuating cycle that substantially contributes to cardiovascular and all-cause mortality. In this context, mortality risk is driven less by modifiable dietary patterns and more by systemic inflammatory and catabolic processes intrinsic to uremia. Therefore, dietary strategies such as DASH, which primarily target metabolic and blood pressure pathways, may have limited capacity to counteract the dominant pathophysiological drivers of death in CKD. This may partially explain why improved dietary adherence did not translate into measurable survival benefits in this subgroup. Based on the above reasons, the net effect of DASH in CKD may be neutral, reflecting a balance between protective metabolic effects and CKD-specific pathophysiologic burdens. Our findings echo the recommendations of the 2020 KDOQI Clinical Practice Guideline for Nutrition in CKD [12], which emphasize that dietary management in CKD should be individualized according to kidney function, metabolic status, and the risk of electrolyte imbalance.

An additional noteworthy finding was the differing association between sodium intake and CV mortality according to CKD status. In participants without CKD, higher sodium intake was associated with increased CV mortality, consistent with established evidence linking excessive sodium consumption to hypertension, vascular dysfunction, and adverse cardiometabolic profiles. In the general population, elevated sodium intake frequently reflects greater consumption of processed and energy-dense foods, which may contribute to overall CV risk. In contrast, this association was not observed among participants with CKD. Several explanations may account for this discrepancy. First, sodium intake in CKD may be influenced by medical counseling, dietary restrictions, reduced appetite, or comorbidity-related dietary modification, potentially introducing residual confounding or reverse epidemiology. Lower sodium intake in CKD may also reflect underlying frailty or poorer nutritional status rather than a protective dietary pattern. Second, CV mortality in CKD is often driven by uremia-related mechanisms, including vascular calcification, myocardial remodeling, volume overload, and arrhythmia, which may attenuate the relative contribution of sodium intake alone. Finally, NHANES dietary assessment relies on 24-hour dietary recalls and does not allow detailed characterization of sodium sources (e.g., processed versus home-prepared foods). Therefore, we were unable to distinguish whether sodium intake reflected unhealthy dietary patterns or necessary caloric intake in CKD patients. Future studies incorporating more granular dietary data and objective measures of sodium exposure are warranted to clarify these differential associations.

This study has several limitations. First, data on patients’ nutritional status—such as serum albumin levels or subjective global assessment—were not available. Second, the definition of CKD relied solely on eGFR, without incorporating albuminuria. Third, the cross-sectional design of this study precludes any inference on causality or long-term outcomes. Nonetheless, this study provides the first evidence linking adherence to the DASH diet with mortality risk in patients with advanced CKD, highlighting its potential clinical relevance. Finally, as with all 24-hour recall–based assessments, measurement error and day-to-day variation in dietary intake are possible and may attenuate observed associations.

## Conclusion

Greater adherence to the DASH diet is associated with lower CV and cancer-related mortality among individuals without CKD. In CKD patients, the benefits are less clear, and some nutrients may have differential effects. Personalized dietary strategies based on kidney function may be warranted.

## Supplementary Information

Supplementary Material 1.

## Data Availability

Data Availability: All relevant data are within the paper and its Supporting Information files.
