# Supplementary material for: Association between DASH diet adherence and mortality in non-diabetic adults with and without chronic kidney disease
Source: Nutr Metab (Lond). 2026 Apr 9;23:59. doi: 10.1186/s12986-026-01118-z (PMC13185240; doi:10.1186/s12986-026-01118-z)

**Supplementary data**

Table 1. Definition of the DASH Score Components

Table 2. Joint effects of adherence to DASH and baseline CKD status on mortality

Table 3. Associations of individual food components of the DASH diet with CV mortality in NHANES participants

Table 4. Table. Associations of individual food components of the DASH diet with Cancer mortality in NHANES participants.

Table 5. Associations of individual food components of the DASH diet with all-cause mortality in NHANES participants.

Figure 1. Scaled Schoenfeld residual plots for the evaluation of the proportional hazards assumption

1A. All-cause mortality

1B. CV mortality

1C. Cancer mortality

1D. For CV or cancer related mortality

Table 1. Definition of the DASH Score Components

| **Component** | **Target for 1 Point** | **Intermediate (0.5 Point)** | **0 Point** |
| --- | --- | --- | --- |
| Total fat (% energy) | ≤ 27% | >27–30% | >30% |
| Saturated fat (% energy) | ≤ 6% | >6–10% | >10% |
| Protein (% energy) | 18% | 16–18% or 18–20% | <16% or >20% |
| Cholesterol (mg/day) | ≤ 150 | 150–300 | >300 |
| Fiber (g/day) | ≥ 30 | 20–29 | <20 |
| Magnesium (mg/day) | ≥ 500 | 250–499 | <250 |
| Calcium (mg/day) | ≥ 1240 | 800–1239 | <800 |
| Potassium (mg/day) | ≥ 4700 | 2400–4699 | <2400 |
| Sodium (mg/day) | ≤ 1500 | 1500–2300 | >2300 |

Table 2. Joint effects of adherence to DASH and baseline CKD status on mortality

|  | Adjusted HR (95% CI)^a^ | P | P for interaction |
| --- | --- | --- | --- |
| **all-cause mortality** | | | |
| DASH score <2 and CKD (-) | 1 (ref) |  |  |
| DASH score <2 and CKD (+) | 1.477(1.342-1.626) | <0.0001 | 0.7652 |
| DASH score >2 and CKD (-) | 0.938(0.871-1.01) | 0.0877 |  |
| DASH score >2 and CKD (+) | 1.413(1.276-1.565) | <0.0001 |  |
| **CV mortality** | | | |
| DASH score <2 and CKD (-) | 1 (ref) |  |  |
| DASH score <2 and CKD (+) | 1.667(1.405-1.976) | <0.0001 | 0.7841 |
| DASH score >2 and CKD (-) | 0.909(0.787-1.05) | 0.1934 |  |
| DASH score >2 and CKD (+) | 1.565(1.335-1.833) | <0.0001 |  |
| **Cancer mortality** | | | |
| DASH score <2 and CKD (-) | 1 (ref) |  |  |
| DASH score <2 and CKD (+) | 0.936(0.732-1.197) | 0.5952 | 0.4149 |
| DASH score >2 and CKD (-) | 0.884(0.753-1.038) | 0.1326 |  |
| DASH score >2 and CKD (+) | 0.948(0.728-1.235) | 0.6891 |  |
| **CV or Cancer mortality** | | | |
| DASH score <2 and CKD (-) | 1 (ref) |  |  |
| DASH score <2 and CKD (+) | 1.371(1.195-1.574) | <0.0001 | 0.4980 |
| DASH score >2 and CKD (-) | 0.898(0.809-0.997) | 0.0443 |  |
| DASH score >2 and CKD (+) | 1.317(1.157-1.499) | <0.0001 |  |

DASH, Dietary Approaches to Stop Hypertension.

^a^ Adjusted for age, gender, body mass index, race, hypertension, and daily energy intake

Table 3. Associations of individual food components of the DASH diet with **CV mortality** in NHANES participants

|  | Overall | | | CKD (-) | | | CKD (+) |  | P for interaction |
| --- | --- | --- | --- | --- | --- | --- | --- | --- | --- |
|  | Adjusted HR  (95% CI)^a^ | | *p* | Adjusted HR  (95% CI)^a^ | *p* | Adjusted HR  (95% CI)^a^ | | *p* |  |
| Component of DASH |  |  | |  |  |  | |  |  |
| Saturated fat 0.5 v.s. 0 | 0.905(0.765-1.069) | 0.2373 | | 0.897(0.744-1.081) | 0.2487 | 0.918(0.638-1.321) | | 0.6423 | 0.6263 |
| 1 v.s. 0 | 0.992(0.807-1.219) | 0.9374 | | 1.045(0.825-1.324) | 0.7106 | 0.902(0.569-1.431) | | 0.6583 |  |
| Total fat 0.5 v.s. 0 | 0.971(0.787-1.198) | 0.7819 | | 0.982(0.779-1.238) | 0.8781 | 0.945(0.638-1.401) | | 0.7765 | 0.8185 |
| 1 v.s. 0 | 0.993(0.823-1.198) | 0.9404 | | 0.985(0.808-1.201) | 0.8783 | 1.054(0.723-1.536) | | 0.7827 |  |
| Protein 0.5 v.s. 0 | 0.921(0.703-1.206) | 0.5453 | | 0.959(0.696-1.321) | 0.7948 | 0.817(0.518-1.289) | | 0.381 | 0.2177 |
| 1 v.s. 0 | 0.921(0.814-1.043) | 0.1923 | | 0.973(0.841-1.126) | 0.7152 | 0.765(0.569-1.029) | | 0.0762 |  |
| Cholesterol 0.5 v.s. 0 | 0.948(0.777-1.156) | 0.5926 | | 0.968(0.762-1.23) | 0.7898 | 0.893(0.593-1.344) | | 0.5827 | 0.8922 |
| 1 v.s. 0 | 1.072(0.927-1.24) | 0.3442 | | 1.084(0.898-1.31) | 0.3952 | 1.105(0.816-1.498) | | 0.5137 |  |
| Fiber 0.5 v.s. 0 | 0.739(0.568-0.961) | 0.0245 | | 0.77(0.575-1.031) | 0.079 | 0.641(0.372-1.107) | | 0.1096 | 0.9212 |
| 1 v.s. 0 | 0.697(0.513-0.946) | 0.0212 | | 0.699(0.497-0.984) | 0.0401 | 0.738(0.424-1.286) | | 0.2801 |  |
| Magnesium 0.5 v.s. 0 | 0.571(0.428-0.762) | 0.0002 | | 0.61(0.446-0.836) | 0.0024 | 0.442(0.196-0.997) | | 0.0491 | 0.7943 |
| 1 v.s. 0 | 0.827(0.605-1.13) | 0.2297 | | 0.845(0.589-1.214) | 0.3591 | 0.79(0.452-1.382) | | 0.4042 |  |
| Calcium 0.5 v.s. 0 | 1.031(0.812-1.31) | 0.7987 | | 1.146(0.884-1.486) | 0.2997 | 0.636(0.383-1.054) | | 0.0786 | 0.5600 |
| 1 v.s. 0 | 0.779(0.636-0.953) | 0.016 | | 0.748(0.593-0.943) | 0.0145 | 0.945(0.641-1.393) | | 0.773 |  |
| Potassium 0.5 v.s. 0 | 0.838(0.69-1.019) | 0.076 | | 0.876(0.702-1.094) | 0.2404 | 0.729(0.453-1.176) | | 0.1924 | 0.7091 |
| 1 v.s. 0 | 0.718(0.563-0.914) | 0.0077 | | 0.743(0.568-0.972) | 0.0306 | 0.624(0.373-1.042) | | 0.0708 |  |
| Sodium 0.5 v.s. 0 | 1.121(0.885-1.418) | 0.3394 | | 1.177(0.865-1.601) | 0.2959 | 0.866(0.572-1.31) | | 0.4907 | 0.0507 |
| 1 v.s. 0 | 1.217(0.976-1.518) | 0.0803 | | 1.324(1.034-1.695) | 0.0266 | 0.873(0.601-1.268) | | 0.4718 |  |

DASH, Dietary Approaches to Stop Hypertension; CV: cardiovascular; HR: hazard ratio; MUFA: monounsaturated fatty acid; SFA: saturated fatty acid. a Adjusted for age, gender, body mass index, race, hypertension, and daily energy intake

CI: confidence interval; HR: hazards ratio

Detailed cut-off value in supplementary table 1.

Table 4. Table. Associations of individual food components of the DASH diet with **cancer mortality** in NHANES participants.

|  | Overall | | | CKD (-) | | CKD (+) |  | P for interaction |
| --- | --- | --- | --- | --- | --- | --- | --- | --- |
|  | Adjusted HR  (95% CI)^a^ | *p* | | Adjusted HR  (95% CI)^a^ | *p* | Adjusted HR  (95% CI)^a^ | *p* |  |
| Component of DASH |  |  |  | |  |  |  |  |
| Saturated fat 0.5 v.s. 0 | 0.876(0.722-1.063) | 0.1762 | 0.849(0.688-1.048) | | 0.1256 | 1.077(0.723-1.605) | 0.7115 | 0.9391 |
| 1 v.s. 0 | 0.888(0.665-1.188) | 0.4201 | 0.898(0.655-1.232) | | 0.5024 | 0.816(0.42-1.585) | 0.5453 |  |
| Total fat 0.5 v.s. 0 | 0.912(0.723-1.151) | 0.434 | 0.846(0.644-1.112) | | 0.2275 | 1.346(0.822-2.205) | 0.2343 | 0.4351 |
| 1 v.s. 0 | 0.937(0.795-1.106) | 0.4394 | 0.922(0.764-1.113) | | 0.3951 | 1.038(0.688-1.565) | 0.8577 |  |
| Protein 0.5 v.s. 0 | 0.787(0.595-1.041) | 0.0919 | 0.756(0.553-1.035) | | 0.0806 | 0.997(0.483-2.057) | 0.9932 | 0.1149 |
| 1 v.s. 0 | 0.913(0.769-1.085) | 0.2978 | 0.882(0.735-1.059) | | 0.1766 | 1.067(0.754-1.509) | 0.7127 |  |
| Cholesterol 0.5 v.s. 0 | 1.004(0.782-1.288) | 0.9762 | 0.979(0.753-1.273) | | 0.8737 | 1.096(0.658-1.826) | 0.7213 | 0.5942 |
| 1 v.s. 0 | 1.114(0.919-1.351) | 0.266 | 1.138(0.929-1.396) | | 0.2089 | 0.961(0.645-1.431) | 0.8419 |  |
| Fiber 0.5 v.s. 0 | 0.676(0.513-0.889) | 0.0056 | 0.641(0.47-0.873) | | 0.0053 | 0.98(0.535-1.796) | 0.9483 | 0.0536 |
| 1 v.s. 0 | 0.564(0.407-0.781) | 0.0007 | 0.508(0.355-0.727) | | 0.0003 | 1.337(0.633-2.828) | 0.4425 |  |
| Magnesium 0.5 v.s. 0 | 0.591(0.448-0.779) | 0.0003 | 0.558(0.416-0.748) | | 0.0001 | 0.998(0.481-2.068) | 0.9951 | 0.0023 |
| 1 v.s. 0 | 0.773(0.562-1.064) | 0.1128 | 0.667(0.471-0.944) | | 0.0228 | 2.285(1.256-4.156) | **0.0073** |  |
| Calcium 0.5 v.s. 0 | 0.7(0.523-0.936) | 0.0168 | 0.711(0.516-0.979) | | 0.037 | 0.551(0.299-1.016) | 0.0561 | 0.2569 |
| 1 v.s. 0 | 0.834(0.654-1.064) | 0.1424 | 0.8(0.616-1.039) | | 0.0939 | 1.153(0.715-1.859) | 0.5565 |  |
| Potassium 0.5 v.s. 0 | 0.738(0.563-0.966) | 0.0273 | 0.686(0.524-0.899) | | 0.0067 | 1.359(0.643-2.872) | 0.4184 | **0.0123** |
| 1 v.s. 0 | 0.758(0.559-1.027) | 0.0731 | 0.678(0.492-0.934) | | 0.0179 | 1.841(1.029-3.296) | **0.0401** |  |
| Sodium 0.5 v.s. 0 | 1.023(0.804-1.301) | 0.8515 | 1.05(0.791-1.392) | | 0.7348 | 0.896(0.556-1.444) | 0.6484 | 0.5920 |
| 1 v.s. 0 | 1.367(1.131-1.654) | 0.0015 | 1.399(1.126-1.739) | | 0.0028 | 1.147(0.701-1.876) | 0.5823 |  |

DASH, Dietary Approaches to Stop Hypertension; CV: cardiovascular; HR: hazard ratio; MUFA: monounsaturated fatty acid; SFA: saturated fatty acid. a Adjusted for age, gender, body mass index, race, hypertension, and daily energy intake

CI: confidence interval; HR: hazards ratio

Detailed cut-off value in supplementary table 1.

Table 5. Associations of individual food components of the DASH diet with **all-cause mortality** in NHANES participants.

|  | | | | | | | | | |
| --- | --- | --- | --- | --- | --- | --- | --- | --- | --- |
|  | Overall | | | CKD (-) | | | CKD (+) |  | P for interaction |
|  | Adjusted HR  (95% CI)^a^ | *p* | | Adjusted HR  (95% CI)^a^ | | *p* | Adjusted HR  (95% CI)^a^ | *p* |  |
| Component of DASH |  | |  | |  |  |  |  |  |
| Saturated fat 0.5 v.s. 0 | 0.892(0.82-0.97) | | 0.0083 | | 0.893(0.808-0.987) | 0.0267 | 0.886(0.73-1.077) | 0.221 | 0.9271 |
| 1 v.s. 0 | 0.899(0.8-1.01) | | 0.0738 | | 0.901(0.796-1.021) | 0.101 | 0.965(0.749-1.242) | 0.7785 |  |
| Total fat 0.5 v.s. 0 | 0.919(0.817-1.034) | | 0.1589 | | 0.935(0.814-1.072) | 0.3309 | 0.863(0.717-1.039) | 0.1176 | 0.6390 |
| 1 v.s. 0 | 0.979(0.9-1.065) | | 0.616 | | 0.969(0.883-1.062) | 0.4938 | 1.048(0.878-1.25) | 0.5995 |  |
| Protein 0.5 v.s. 0 | 0.942(0.814-1.091) | | 0.4203 | | 0.912(0.77-1.08) | 0.2828 | 1.066(0.852-1.334) | 0.5745 | 0.1987 |
| 1 v.s. 0 | 0.956(0.887-1.029) | | 0.2281 | | 0.983(0.903-1.069) | 0.6821 | 0.828(0.704-0.974) | 0.0233 |  |
| Cholesterol 0.5 v.s. 0 | 0.97(0.859-1.095) | | 0.619 | | 0.962(0.834-1.109) | 0.5904 | 0.994(0.793-1.247) | 0.9587 | 0.6688 |
| 1 v.s. 0 | 0.997(0.905-1.097) | | 0.9426 | | 0.992(0.888-1.108) | 0.8841 | 1.062(0.881-1.28) | 0.5236 |  |
| Fiber 0.5 v.s. 0 | 0.714(0.627-0.814) | | **<0.0001** | | 0.692(0.598-0.8) | **<0.0001** | 0.866(0.655-1.145) | 0.31 | **0.0221** |
| 1 v.s. 0 | 0.679(0.602-0.765) | | **<0.0001** | | 0.644(0.565-0.734) | **<0.0001** | 0.999(0.749-1.334) | 0.9968 |  |
| Magnesium 0.5 v.s. 0 | 0.683(0.588-0.793) | | **<0.0001** | | 0.681(0.586-0.792) | **<0.0001** | 0.78(0.538-1.131) | 0.1878 | 0.1687 |
| 1 v.s. 0 | 0.766(0.668-0.877) | | **0.0002** | | 0.742(0.638-0.863) | 0.**0002** | 0.952(0.728-1.246) | 0.7176 |  |
| Calcium 0.5 v.s. 0 | 0.926(0.809-1.06) | | 0.2594 | | 0.937(0.804-1.092) | 0.3999 | 0.842(0.621-1.144) | 0.2682 | 0.0835 |
| 1 v.s. 0 | 0.916(0.814-1.032) | | 0.1464 | | 0.879(0.767-1.006) | 0.0606 | 1.187(0.929-1.516) | 0.1673 |  |
| Potassium 0.5 v.s. 0 | 0.823(0.721-0.939) | | **0.0044** | | 0.806(0.696-0.934) | **0.0047** | 0.997(0.753-1.32) | 0.9824 | 0.4153 |
| 1 v.s. 0 | 0.805(0.709-0.915) | | **0.0011** | | 0.794(0.688-0.916) | **0.0019** | 0.914(0.701-1.191) | 0.4997 |  |
| Sodium 0.5 v.s. 0 | 1.124(1.021-1.237) | | **0.0178** | | 1.184(1.047-1.337) | **0.0074** | 0.859(0.667-1.106) | 0.2349 | 0.0595 |
| 1 v.s. 0 | 1.322(1.199-1.458) | | **<0.0001** | | 1.371(1.228-1.532) | **<0.0001** | 1.068(0.867-1.316) | 0.534 |  |

DASH, Dietary Approaches to Stop Hypertension; CV: cardiovascular; HR: hazard ratio; MUFA: monounsaturated fatty acid; SFA: saturated fatty acid.

a Adjusted for age, gender, body mass index, race, hypertension, and daily energy intake

CI: confidence interval; HR: hazards ratio

Detailed cut-off value in supplementary table 1.

Figure 1. Scaled Schoenfeld residual plots for the evaluation of the proportional hazards assumption

1A. All-cause mortality


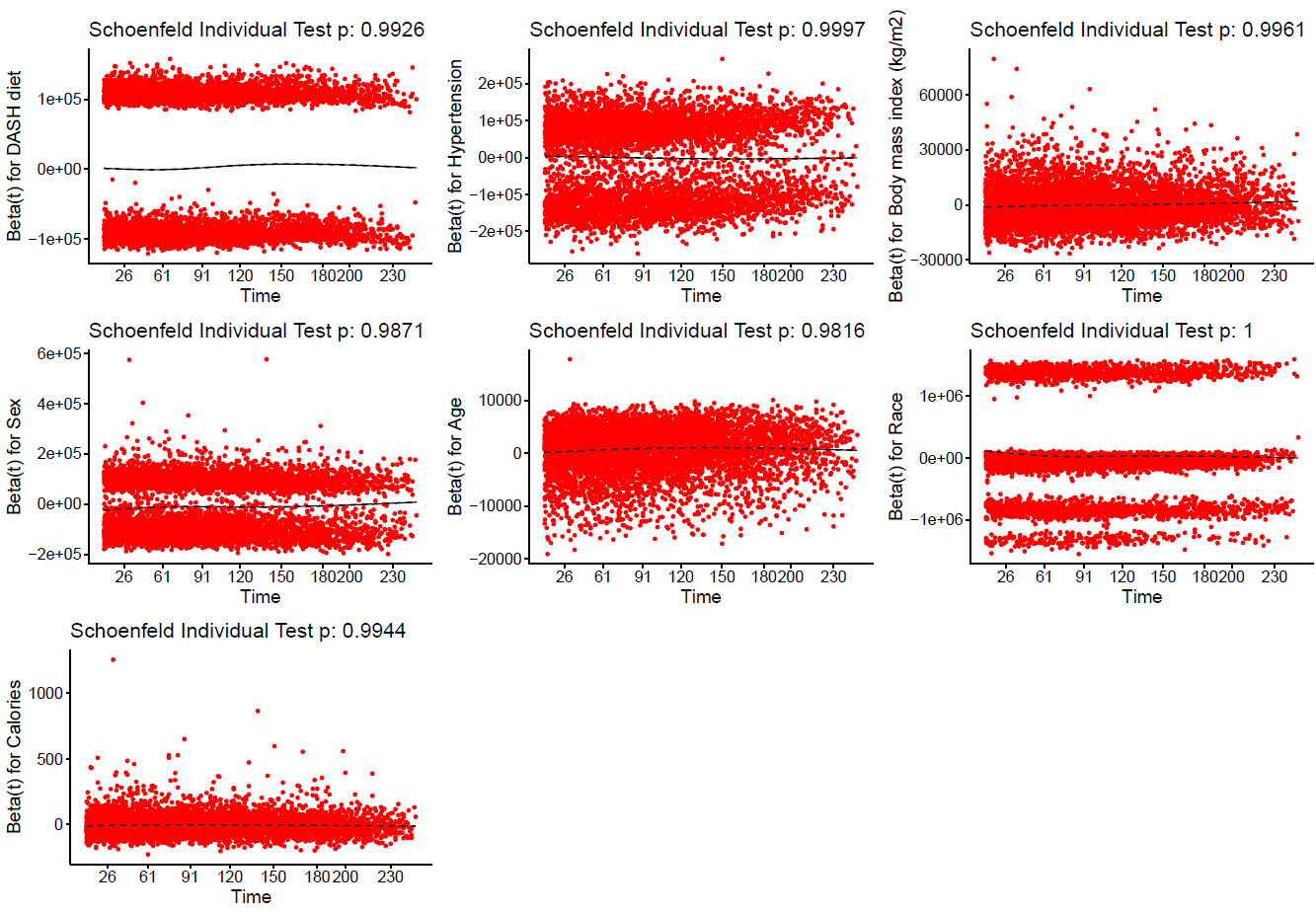


1B. CV mortality


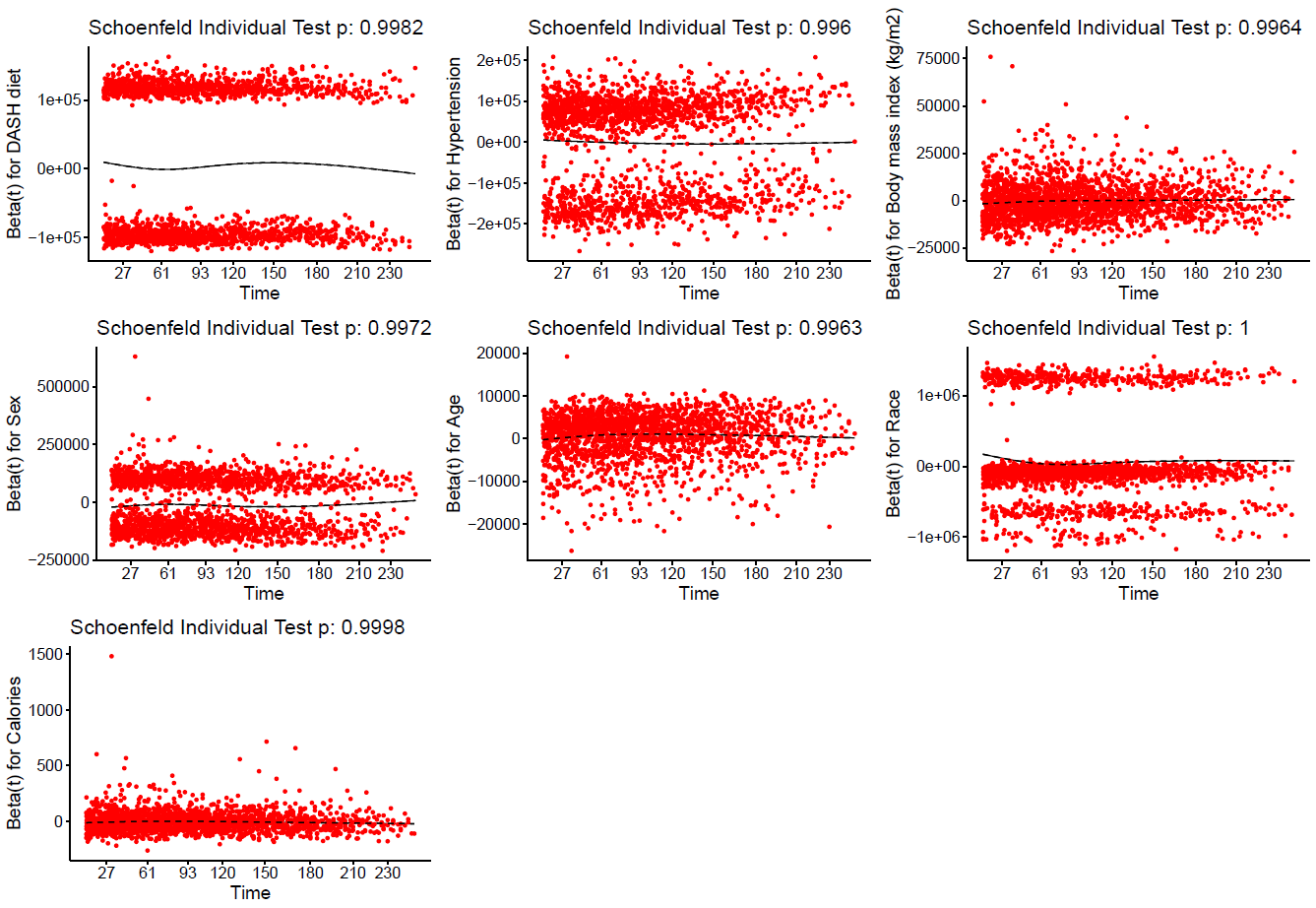


1C. Cancer mortality


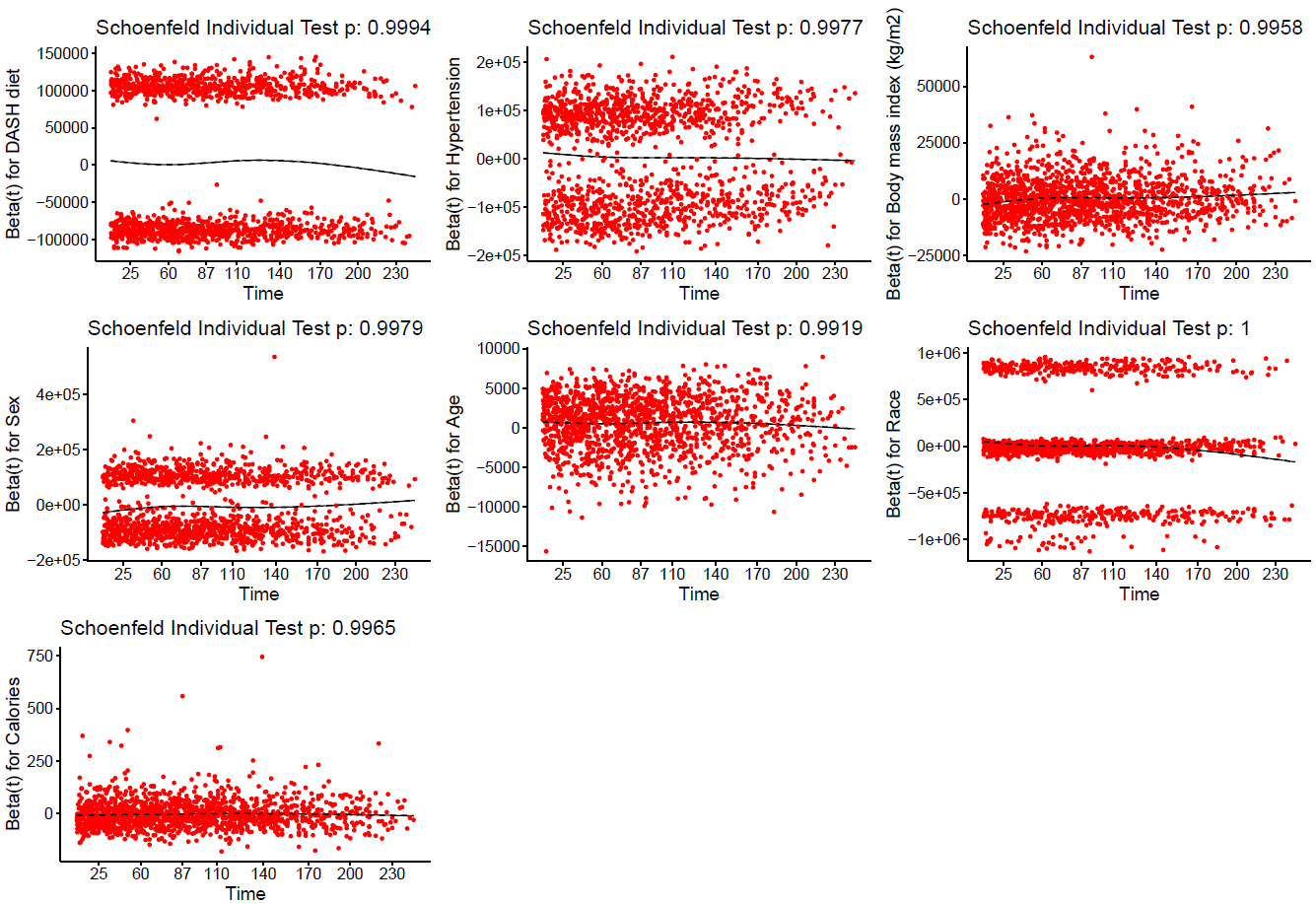


1D. For CV or cancer related mortality


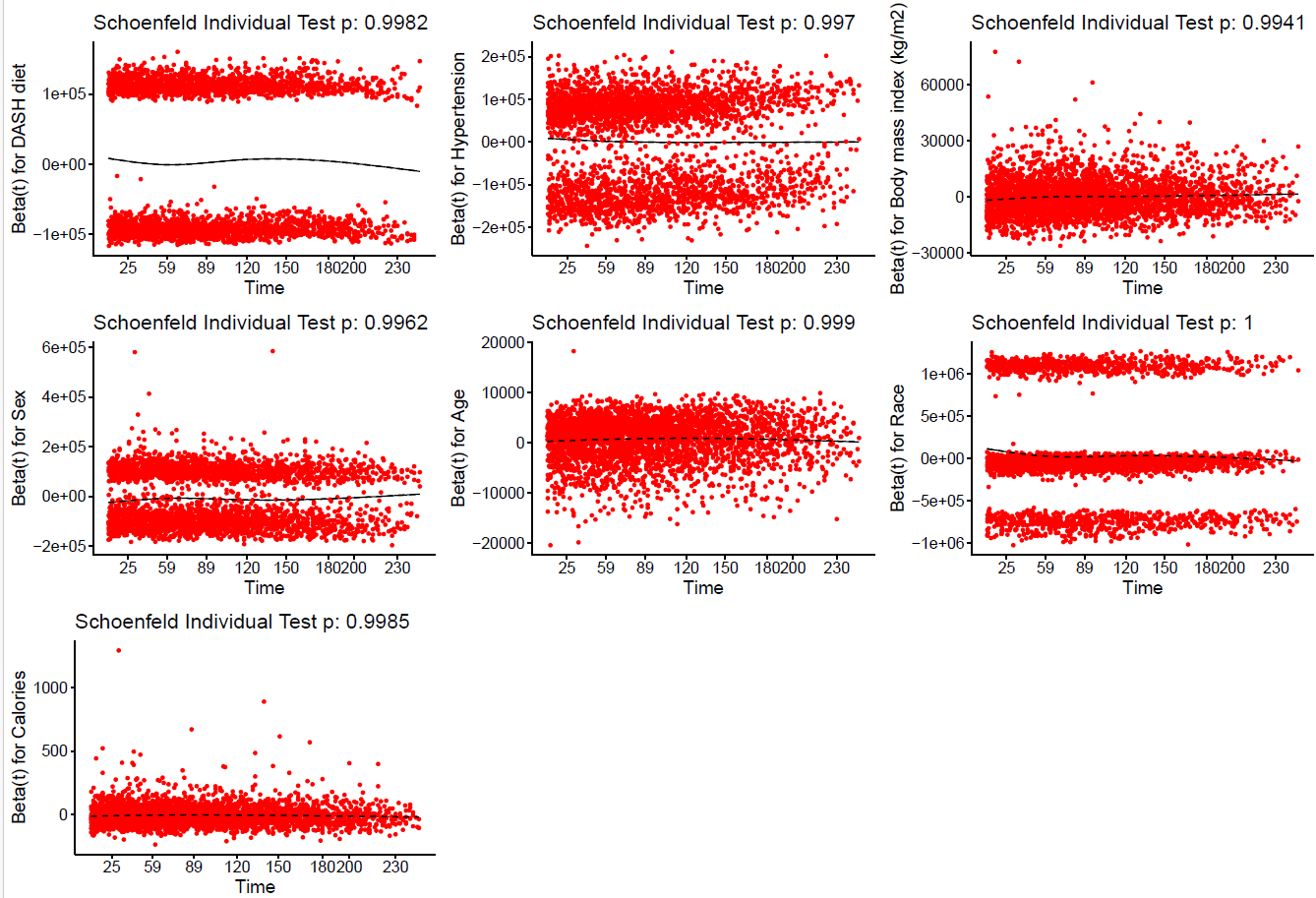

Supplement: Supplementary file 1 — Supplementary Material 1. [file 12986_2026_1118_MOESM1_ESM.docx]
